# GVC: efficient random access compression for gene sequence variations

**DOI:** 10.1186/s12859-023-05240-0

**Published:** 2023-03-28

**Authors:** Yeremia Gunawan Adhisantoso, Jan Voges, Christian Rohlfing, Viktor Tunev, Jens-Rainer Ohm, Jörn Ostermann

**Affiliations:** 1 Institut für Informationsverarbeitung and L3S Research Center, Leibniz University Hannover, Hannover, Germany; 2 Institut für Nachrichtentechnik, RWTH Aachen University, Aachen, Germany

**Keywords:** Variants, VCF, Compression, Random access

## Abstract

**Background:**

In recent years, advances in high-throughput sequencing technologies have enabled the use of genomic information in many fields, such as precision medicine, oncology, and food quality control. The amount of genomic data being generated is growing rapidly and is expected to soon surpass the amount of video data. The majority of sequencing experiments, such as genome-wide association studies, have the goal of identifying variations in the gene sequence to better understand phenotypic variations. We present a novel approach for compressing gene sequence variations with random access capability: the Genomic Variant Codec (GVC). We use techniques such as binarization, joint row- and column-wise sorting of blocks of variations, as well as the image compression standard JBIG for efficient entropy coding.

**Results:**

Our results show that GVC provides the best trade-off between compression and random access compared to the state of the art: it reduces the genotype information size from 758 GiB down to 890 MiB on the publicly available 1000 Genomes Project (phase 3) data, which is 21% less than the state of the art in random-access capable methods.

**Conclusions:**

By providing the best results in terms of combined random access and compression, GVC facilitates the efficient storage of large collections of gene sequence variations. In particular, the random access capability of GVC enables seamless remote data access and application integration. The software is open source and available at https://github.com/sXperfect/gvc/.

## Introduction

In the course of the last decades, the cost of genome sequencing has dropped significantly, to less than USD 1000, enabling the use of genomic information in many fields, such as precision medicine, oncology, and food quality control. This led to an explosion in data generation, with the volume of data generated annually soon surpassing that of other big data domains such as video and astronomy [1].

A major focus of genetic research is the analysis of gene sequence variations. In fact, the discovery of gene sequence variations among large populations of related samples is one of the main applications of next- and third-generation sequencing technologies. Genetic variations can be classified into: (i) single-nucleotide polymorphisms (SNPs); (ii) insertions and deletions (indels); (iii) (large) structural variants (SV). Such gene sequence variations (or “variants”) are commonly stored in the text-based variant call format (VCF) [2].

A VCF file consists of two main parts: the header and the actual variant records. Each variant record is stored on a single line and separated into multiple annotations, which can be either site-level or sample-level.

Regarding the site-level annotations, the first eight columns (CHROM, POS, ID, REF, ALT, QUAL, FILTER, INFO) represent the properties observed at the variant site. When multiple samples are represented in a VCF file, some of the site-level annotations represent a summary or average of the values obtained for that site from different samples. The following site-level annotations are relevant in the scope of our work: CHROM—the contig (usually a chromosome) on which the variant occurs; POS—the 1-based genomic coordinate on the contig of the variant (note that for deletions the position given is the base preceding the event); REF—the reference allele; ALT—the alternative allele(s) observed in a sample, or a set of samples (note that REF and ALT are given on the forward strand, for insertions, the ALT allele includes the inserted sequence as well as the base preceding the insertion, for deletions, the ALT allele is the base before the deletion). Sample-level annotations are contained in the 9th column (FORMAT) and in the sample columns (10th and beyond). Sample-level annotations are tag-value pairs. The tags are recorded in the FORMAT field. The values are recorded in the corresponding order in each sample column.

Our work focuses on the sample-level annotation with the GT tag. Its value indicates the genotype of a sample at a variant site. For a diploid organism, the value indicates the two alleles carried by the sample, encoded by a 0 for the REF allele, 1 for the first ALT allele, 2 for the second ALT allele, and so on. In addition, phasing information is incorporated to each genotype value: “/” for unphased genotype and “|” for phased genotype. For example, if there is a single ALT allele (which is by far the most common case), the value will be either: 0/0—the sample is homozygous reference; 0/1—the sample is heterozygous, carrying one copy each of the REF and ALT alleles; 1/1—the sample is homozygous alternate.

For non-diploids, the same pattern applies; in the haploid case, there will be just a single number; for polyploids, there will be more, e.g., 4 numbers for a tetraploid organism.

In summary, VCF files contain the actual variant information together with a considerable amount of metadata pertaining to the variant calling process. The metadata is primarily used to filter out irrelevant variants. Once the variants are filtered, the genotype and phasing information associated with each sample generally becomes the primary information for further downstream analyses.

In this paper, we introduce a novel approach for compressing gene sequence variations with random access capability: the Genomic Variant Codec (GVC). We compare our approach with three existing approaches: GTRAC [3], GTC [4], and GTShark [5]. GTRAC, introduced in 2016, is based on a customized Lempel-Ziv compressor [6]. In 2018, Danek et al. introduced column-wise sorting (i.e., a permutation of haplotypes) in GTC to minimize the number of differences between adjacent samples. They use a combination of techniques such as run-length encoding, Lempel-Ziv representation, and Huffman coding for compression. Both GTRAC and GTC offer random access capability. GTShark, proposed by Deorowicz et al. in 2019, achieves higher compression using a combination of generalized version of positional Burrows-Wheeler transform (PBWT) [7] called gPBWT [8] and a range coder [9] with special contextual modeling. The Burrow-Wheeler transform (BWT) rearranges the symbols by permuting the order of the characters based on their lexicographic order. This results in runs of similar characters. In comparison, both PBWT and gPBWT permute the rows based on the previous rows. This maximizes the compression efficiency because the genotypes of adjacent locations or positions tend to be highly correlated. Range coding is an entropy coding method that works by dividing a large range of integers, representing an interval [0, 1). The range is divided into sub-ranges whose sizes are proportional to the probability of the symbol they represent. The input symbol is then mapped to an interval, in which the corresponding probability lies.

Since the goal of GTShark is to maximize compression, the intermediate sample permutations are not stored and thus random access is not supported. However, in principle, the concept of GTShark does not prohibit implementing random access.

## Methods

**Fig. 1:**
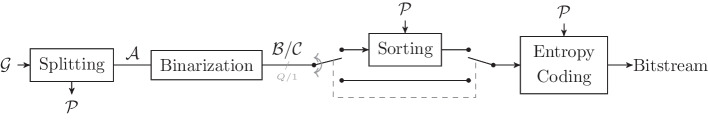
Block diagram of the proposed encoding process. The genotype matrix $\mathcal {G}$ is processed by a series of transformations: splitting, binarization, and optionally sorting. At the end of the process, entropy coding is applied

We propose a new tool called Genomic Variant Codec (GVC) for the compression of gene sequence variations. GVC comprises transformations and entropy coding steps depicted in Fig. 1.

In our proposed approach, the genotypes are extracted from a VCF file and divided into blocks. Each block $\mathcal {G}$ represents genotypes of all samples in a certain range of loci in a chromosome (or across multiple chromosomes). Splitting into blocks allows for non-sequential access and parallel processing. Each block is then further divided into two different matrices: the allele matrix $\mathcal {A}$ and the phasing matrix $\mathcal {P}$. The phasing matrix is a binary matrix while the allele matrix is an integer matrix.

We propose two alternative binarization approaches to decompose the allele matrix into a binary representation: bit plane binarization and row binarization. These steps allow for variable bit-lengths for integer values. The choice between bit plane binarization and row binarization depends on the properties of the data. Bit plane binarization yields bit planes $\mathcal {B}_q$ that can be concatenated into a single binary matrix $\mathcal {C}$. Alternatively, row binarization yields only a single binary matrix.

Subsequently, optionally, the row- and column-wise sorting is applied to binary matrices. The motivation behind the sorting process is to maximize the average run-length of zeros and ones in both directions, which then facilitates more efficient entropy coding. We use the Hamming distance to measure the similarity between adjacent rows and columns. At the end of the process, each binary matrix is entropy-encoded. In our experiments, we used an implementation of the JBIG standard, which specifies bi-level image compression [10]. Note that, in principle, any other entropy codec could be used for this purpose. In the following sections, we explain the four stages of GVC—splitting, binarization, sorting, and entropy coding—in more detail.

### Splitting

**Fig. 2:**
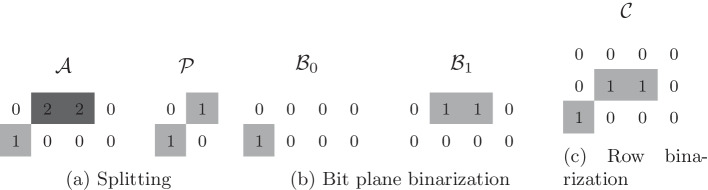
Example for $${\mathcal {G}} = \begin{array}{ll} 0\mid 2 &{} 2/0 \\ 1/0 &{} 0 \mid 0 \end{array}$$. Bit plane binarization yields two bit planes representing the most significant bit and the less significant bit of $\mathcal {A}$. Row binarization generates only three binary rows because the first row of $\mathcal {A}$ requires two bits and the second row of $\mathcal {A}$ requires only one bit

In the first step, the genotypes are extracted from a VCF file and divided into blocks. We also refer to each block as a genotype matrix $\mathcal {G}$. The genotype matrix is then further decomposed into an allele matrix and a phasing matrix as shown in Fig. 2a. Given *m* variant records, each containing GT annotations for *n* *p*-ploid samples, the allele matrix $\mathcal {A}$ can be formulated as follows:

1 $$\begin{aligned} \mathcal {A} {:}{=}\begin{pmatrix} a_{0,0,0}, \dots , a_{0,0,(p-1)} &{} \dots &{} a_{0,(n-1),0}, \dots , a_{0,(n-1),(p-1)} \\ \vdots &{} \ddots &{} \vdots \\ a_{(m-1),0,0}, \dots , a_{(m-1),0,(p-1)} &{} \dots &{} a_{(m-1),(n-1),0}, \dots , a_{(m-1),(n-1),(p-1)} \end{pmatrix}\text {,} \end{aligned}$$

with $a_{i,j,k} \in \mathbb {N} \cup \{0\}$. Phasing information such as unphased (“/”) and phased (“|”) is stored in the phasing matrix $\mathcal {P}$ as values 0 and 1, respectively. The phasing matrix $\mathcal {P}$ can be formulated as follows:

2 $$\begin{aligned} \mathcal {P} {:}{=}\begin{pmatrix} p_{0,0} &{} \dots &{} p_{0,(n-1)} \\ \vdots &{} \ddots &{} \vdots \\ p_{(m-1),0} &{} \dots &{} p_{(m-1),(n-1)} \end{pmatrix} \end{aligned}$$

with $$p_{i,j}\in \{0,1\}$$.

Below are $m=2$ example variant records, each containing GT annotations for $n=2$ samples named S1 and S2:

$$\begin{aligned} \begin{array}{lllllll} \text {CHROM} \quad \quad &{} \text {POS} \quad \quad &{} \text {REF} \quad \quad &{} \text {ALT} \quad \quad &{} \text {FORMAT} \quad \quad &{} \text {S1} \quad \quad &{} \text {S2}\\ \text {1} \quad \quad &{} \text {1} \quad \quad &{} \text {A} \quad \quad &{} \text {G} \quad \quad &{} \text {GT} \quad \quad &{} \text {0}\vert \text {2} \quad \quad &{} \text {2/0}\\ \text {1} \quad \quad &{} \text {10} \quad \quad &{} \text {C} \quad \quad &{} \text {A} \quad \quad &{} \text {GT} \quad \quad &{} \text {1/0} \quad \quad &{} \text {0}\vert \text {0}.\end{array} \end{aligned}$$

The corresponding $m\times n$ genotype matrix $\mathcal {G}$ is then split into allele matrix $\mathcal {A}$ and phasing matrix $\mathcal {B}$ as follows:

$$\begin{aligned} \mathcal {G} = \begin{pmatrix} 0|2 &{} 2/0\\ 1/0 &{} 0|0 \end{pmatrix} \;\xrightarrow []{\text {split}}\; \mathcal {A} = \begin{pmatrix} 0 &{} 2 &{} 2 &{} 0\\ 1 &{} 0 &{} 0 &{} 0 \end{pmatrix} ,\;\; \mathcal {P} = \begin{pmatrix} 0 &{} 1\\ 1 &{} 0 \end{pmatrix}\text {.} \end{aligned}$$

In the special case that all genotypes are either phased or unphased, it is not necessary to encode and transmit the phasing matrix $\mathcal {P}$. Instead, the information (that all genotypes are (un)phased) can be signaled with a flag. In some cases, allele values may be missing, indicated by a dot (“.”) in the VCF file, or the ploidy may vary along the rows. To handle missing values, we replace the dots with a special integer value. If the ploidy varies within a block, the genotypes are padded with another special integer value so that the ploidy is uniform within a block.

### Binarization

In the next step, the allele matrix $\mathcal {A}$ is binarized. We propose two alternative binarization approaches: bit plane binarization (an example is shown in Fig. 2b) and a binarization using a technique we call “row binarization” (an example is shown in Fig. 2c).

In the bit plane binarization, each value $a_{i,j,k}$ of the allele matrix $\mathcal {A}$ is expressed as a set of binary numbers. All *q*-th significant bits are then aggregated as a bit plane $\mathcal {B}_q$. The number of bit planes *Q* is computed based on the maximum value in the allele matrix $\mathcal {A}$:

3 $$Q = \left\lceil {\log _{2} \left( {\max _{{\forall i,j,k}} \left\{ {a_{{i,j,k}} } \right\} + 1} \right)} \right\rceil.$$

The allele values $a_{i,j,k}$ are expressed as binary numbers using exactly *Q* bits per value and the bit plane binarization yields *Q* bit planes. The *q*-th bit plane $\mathcal {B}_q$ is the concatenation of the *q*-th least significant bit of each allele value, with $q\in \{0,\cdots ,(Q-1)\}$. Optionally, bit planes can be concatenated either row-wise or column-wise.

In the row binarization, each row is decomposed into multiple binary rows as follows. Each row *i* contains allele values $a_{i,j,k}$ with the corresponding maximum allele value being denoted as $a_{i}^{\text {max}}$. Given a maximum allele value $a_{i}^{\text {max}}$, we can compute the number of bits required to represent the row (using a naïve binary encoding) as $R=\left\lceil\log _2\left( a_{i}^{\text {max}}+1\right) \right\rceil$. The number of required bits *R* is equal to the number of resulting binary rows. The *r*-th binary row contains the *r*-th least significant bit of allele values of the respective row. At the end of the process, all of the resulting binary rows are concatenated row-wise, which yields a single binary matrix. Note that the reconstruction of the allele matrix $\mathcal {A}$ from this binary matrix is possible because the maximum allele value $a_{i}^{\text {max}}$ can be computed based on the number of alleles in the ALT column of the original VCF file. Within GVC, this information is communicated to the decoder as side information.

Both bit plane and row binarization have their own advantages and disadvantages. The binarization can be chosen based on the number of alternate alleles of each row within a block. Bit plane binarization is most advantageous when the number of alternate alleles is constant. In such a case, row binarization generates a larger matrix because the number of bits of each row must be preserved, even if the number of alternate alleles is constant. In contrast, row binarization is advantageous when the number of alternate alleles within a block is not constant. For real-world data, we recommend row binarization over bit plane binarization as the default binarization because of the variable number of alternate alleles.

### Sorting

In previous works such as GTC [4], the sorting of haplotype matrix columns was proposed to improve the performance of subsequent entropy coding schemes. In addition to column-wise sorting, here we propose row-wise sorting. Preliminary experiments showed that subsequent entropy coding schemes yield smaller bitstreams when rows are sorted in particular ways. One possible reason lies in the fact that if some samples belong to a group of similar individuals, then the variants found on these samples are expected to be similar. By sorting in both directions, run lengths can be maximized, which presumably also facilitates a more efficient entropy coding. Note that sort indices must be transmitted to reconstruct the original row order.

As an example, consider the following allele matrix $\mathcal {A}$:

$$\begin{aligned} \mathcal {A} = \begin{pmatrix} 0\ 2 &{} 2\ 0\\ 1\ 0 &{} 0\ 0 \end{pmatrix}. \end{aligned}$$

Let us assume that swapping the rows lets a subsequent entropy coding scheme yield a smaller bitstream. The sorted allele matrix $\tilde{\mathcal {A}}$ is then as follows:

$$\begin{aligned} \tilde{\mathcal {A}} = \begin{pmatrix} 1\ 0 &{} 0\ 0\\ 0\ 2 &{} 2\ 0 \end{pmatrix}. \end{aligned}$$

To reconstruct the allele matrix $\mathcal {A}$ at the decoder, the sort row indices ${\tilde{a}} = (1\;0)^T$ must be transmitted.

Before the sorting process, a cost matrix *C* based on the Hamming distance [11] is computed for each pair of rows or columns depending on the sorting direction. The Hamming distance of a pair of rows or columns is the number of positions where the corresponding values differ. The entry of the cost matrix $c_{ij}$ at row *i* and column *j* describes the Hamming distance between the *i*-th and *j*-th rows for row sorting and *i*-th and *j*-th columns for column sorting. The main diagonal of the cost matrix is zero.

The time complexity of computing the cost matrix is quadratic with regard to the block size and the number of samples. To alleviate this limitation, we can also split the genotypes into blocks in the column direction. This reduces the sorting cost and the decoding time. However, this comes at the expense of compression performance.

Note that any other suitable cost function could be used instead (of course, it should ideally be chosen to match the chosen entropy coding method). At the end of the sorting process, the order of rows and/or columns, respectively, which provides the lowest total cost, is selected. This selection process can be regarded as equivalent to solving the traveling salesman problem, which we solve using a nearest neighbor heuristic. Other methods of solving this problem, such as the Lin-Kernighan heuristic [12], could be employed to achieve a potentially better compression at the expense of encoding time.

### Entropy coding

The results of the transformations, i.e., binarizations and sorting procedures, are binary matrices $\mathcal {B}_q$, $\mathcal {C}$, $\mathcal {P}$ which we interpret as binary images. Any method that can efficiently encode binary images losslessly can be utilized for entropy coding, such as binary arithmetic coding. Even better compression performance can be achieved by estimating the source statistics, e.g. using context modeling, which estimates the probability of a current symbol depending on previous symbols. For GVC, we choose an encoder compliant to the JBIG standard (ISO/IEC 11544 [10]). JBIG specifies the lossless compression of bi-level images.^1^ It takes advantage of the spatial correlation of bi-level pixels for efficient compression. The length of the context model in JBIG varies between in total of 10 to 12 neighboring values in both row and column directions, depending on the selected context mode. In combination with the sorting, we maximize the similarity between the adjacent columns or rows of the binary matrices. This generally enables JBIG to infer better context models, thus increasing the overall compression efficiency.

## Results

For our simulations, we used data from phase 3 of the 1000 Genomes Project [14]. The data set consists of 22 VCF files with a total size of 770 GiB. The genotype information occupies the largest share of 758 GiB.

We can configure GVC with several parameters: the binarization can be either bit plane binarization or row binarization; the sorting can be disabled, configured for rows or columns only, or enabled for both rows and columns. We set the block size to 2504 variant records based on empirical experiment. Since we sort both rows and columns, we seek a well-balanced trade-off between the compression improved by the sorting and the overhead induced by transmitting sorting indices. Thus, by assuming that at each variant site there are roughly three alternate alleles, we compute the block size of 2504 by multiplying the number of samples (2504) by the ploidy (2) divided by the average number of bits required to represent each value (2). Also, we fixed the following encoder parameters: the sorting algorithm (nearest neighbor), the sorting metric (Hamming distance), and the compressor (JBIG). In total we ended up with 12 configurations for GVC: $2+1$ binarizations (bit plane with and without concatenation, and row binarization) $\times$ 4 sorting schemes (none, col, row, both). These configurations and their corresponding identifiers are listed in Table 1.

**Table 1:** GVC configurations

ID	Binarization	Sorting	ID	Binarization	Sorting
0	Bit plane (concat.)	None	6	Bit plane	Row
1	Bit plane (concat.)	Col	7	Bit plane	Both
2	Bit plane (concat.)	Row	8	Row	None
3	Bit plane (concat.)	Both	9	Row	Col
4	Bit plane	None	10	Row	Row
5	Bit plane	Col	11	Row	Both

**Fig. 3:**
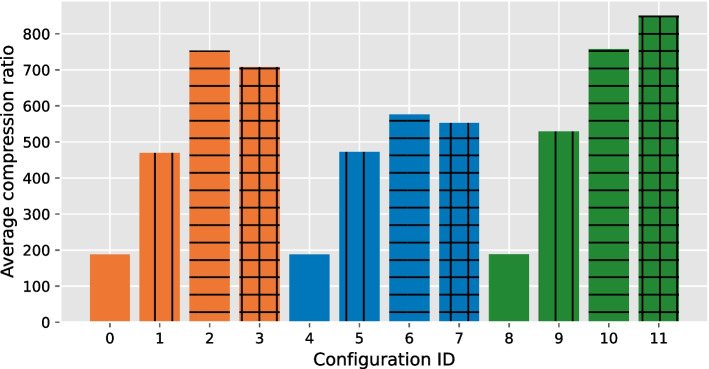
Average compression ratio (averaged over all chromosomes) achieved by each GVC configuration. The colors indicate the employed binarization: orange—bit plane binarization (concatenated), blue—bit plane binarization, green—row binarization. The patterns indicate the employed sorting: no pattern—no sorting, vertical lines—column sorting, horizontal lines—row sorting, both horizontal and vertical lines—sorting in both directions

To evaluate the impact of both the binarization and sorting methods on the overall compression performance of GVC, we compute the average compression ratio, which is computed by dividing the original file size by the compressed file size. We compute the average over all chromosomes for each GVC configuration. Figure 3 shows the corresponding results.

Configuration 11 (row binarization in combination with sorting of both columns and rows) outperforms all other configurations. Note that the average compression ratio is greatly improved by enabling sorting. Especially in the case of row binarization, enabling sorting both row- and column-wise yields the best results. Without concatenation, the bit plane transformation yields multiple bit planes that are sorted independently. This results in a larger overhead due to storing the corresponding sort indices.

When bit plane binarization is selected as the binarization scheme, sorting columns performs slightly better than sorting both columns and rows. This is because bit plane binarization creates a new bit plane even if only one row (corresponding to a single variant site) requires additional bits to correctly represent the allele value. In such case, the number of rows becomes greater than the number of columns; this renders the row sorting inefficient as the overhead induced by sorting indices overcomes the increase in compression. For the row binarization, the number of generated binary rows is less compared to the rows generated by the bit plane binarization. The resulting binary matrix will have a greater number of columns compared to the number of rows. Thus, sorting in column direction induces a greater overhead, resulting in a worse compression ratio.

**Fig. 4:**
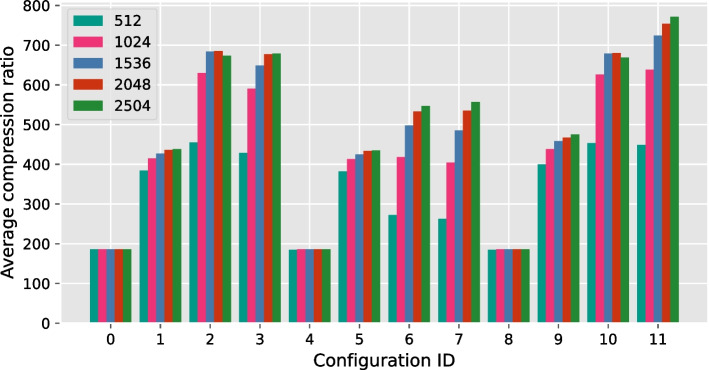
Compression ratio achieved by each GVC configuration with different block sizes. For all configurations with the sorting enabled, increasing the block size increases the compression ratio

We also evaluated the effect of the block size on the compression performance of GVC. We compressed the chromosome 22 file with all the configurations that are listed in Table 1 and five different block sizes: 512, 1024, 1536, 2048, and 2504. The result is shown in Fig. 4. As expected, increasing the block size increases the compression ratio. There are however two expections: for configuration IDs 2 and 10, increasing the block size beyond 2048 leads to a degradation in compression ratio. This is because in these cases the larger number of row sort indices generates an overhead that is not remedied by the gains achieved through the larger block size.

**Fig. 5:**
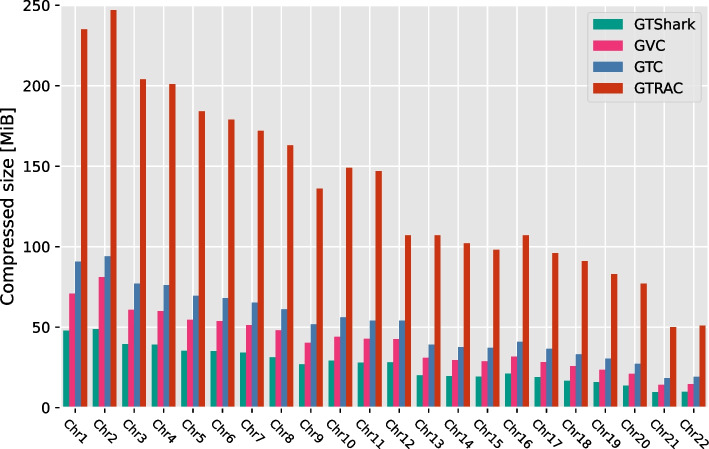
Comparison of GVC to the state-of-the-art methods GTC [4], GTRAC [3], and GTShark [5] with respect to compressed size

Finally, we compare GVC to its competitors GTRAC, GTC, and GTShark. All simulations were performed on a Linux platform with an Intel$^\mathrm{(R)}$ Core$^\mathrm{(TM)}$ i9-9900K CPU, running at 5 GHz, and a solid state drive. For GTRAC, GTC, and GTShark, we used their default parameters; for GVC we used configuration 11 because it yields the best compression ratio. Figure 5 shows the compressed size in MiB for each chromosome for GVC, GTRAC, and GTC.

**Fig. 6:**
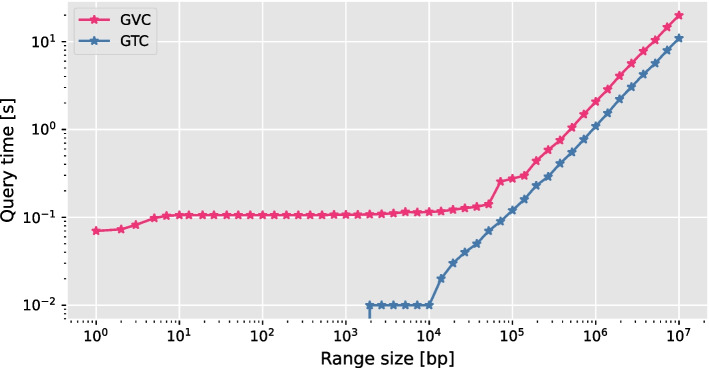
Comparison of random access time between GTC [4] and GVC with respect to the range size

**Fig. 7:**
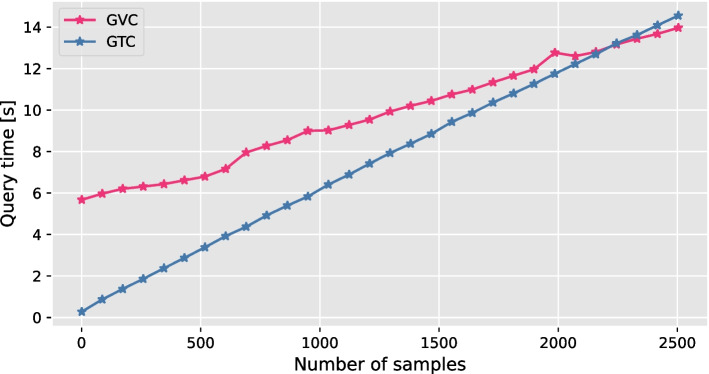
Comparison of random access time between GTC [4] and GVC with respect to the number of samples

With respect to random access, we only compare GVC to GTRAC and GTC, since GTShark does not provide random access functionality. In the case of GTRAC, we were unable to query, i.e., decode, the tested dataset. We refer to the random access time, or query time, as the total time it takes a method to access arbitrary elements in the compressed data. This is done by partially decoding and inverse-transforming the payloads given a query. We analyze the time complexity by varying the number of samples and the number of variants.

First, we compare the query time on all samples and different range sizes of the random access. We refer to this type of query as a range query. For a range query, we decode the genotypes of all samples in a region. Three values specify a region: a chromosome, a start position, and an end position. We refer to the difference between the start and end positions as the range size. Because variants do not occur at every locus, the number of variant sites (i.e., rows) is always smaller or equal than the range size. As presented in Fig. 6, GTC performs about 10x and 2x faster than GVC for smaller and larger range sizes, respectively.

Figure 7 shows the result of the second experiment, where we queried variant sites within a range of $1\textrm{e}{7}$ bp while varying the number of samples. GVC’s JBIG-based decoder decodes whole blocks even if the query requires one variant site or sample, but the inverse transformation is performed only on the queried variant sites or samples. This results in a higher run time overhead for smaller range sizes. For larger range sizes, the complexity of the entropy decoder and the transformation dominate the run time.

**Fig. 8:**
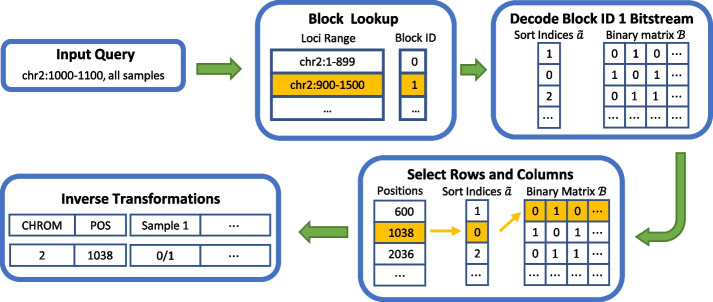
An example of a random access process on compressed genotypes where the number of alternate alleles is one and the blocks are transformed using bit plane binarization and sorted in row direction. A user needs the genotypes of all samples on chromosome 2 at loci 1000 through 1100, represented by “chr2:1000-1100”. First, GVC finds the blocks containing the required genotype information using a block lookup process. The bitstreams of the selected block, in this case the block with ID 1, are then decoded, yielding the sort indices ${\tilde{a}}$ and the binary matrix $\mathcal {B}$. Using the position information of each variant site, GVC selects certain rows or columns of the binary matrix $\mathcal {B}$ and based on the sort index ${\tilde{a}}$. Finally, the selected rows and columns of the binary matrix $\mathcal {B}$ are then inverse transformed to return the genotypes of all samples at loci 1000 through 1100

To better explain this, suppose we have a genotype matrix $\mathcal {A}$ where the number of alternate alleles is one. The matrix is then transformed using bit plane binarization and sorted in the row direction. The bit plane binarization yields only one binary matrix because the largest value of the genotype matrix is one. A user requests genotype information for all samples on chromosome 2 at loci 1000–1100. GVC then performs the random access process shown in Fig. 8. An entire block needs to be entropy decoded (the one with block ID 1), although only a small range (chr2:1000–1100) was requested, and although it even only contains a single variant site (1038).

We believe that random access times below 0.5 s are not noticeable. Note that GVC is mostly written in Python (except for JBIG and the transformation steps which are written in C), whereas GTC is written entirely in C, introducing some overhead to GVC with regard to the run time. This is a subject for further improvements in the future.

Finally, to summarize the results, Table 2 shows the compressed sizes and random access times. The absolute compressed size is given for each method as the sum of the results obtained for each chromosome, as shown in Fig. 5. To evaluate the random access capability, we calculated the area under the curve shown in Fig. 7. In summary, GVC offers a very good trade-off between GTShark and GTC: GVC achieves slightly worse compression results than GTShark while providing random access functionality. GVC outperforms GTC in terms of compression, but gives similar random access times for range-based access and slightly worse for sample-based access.

**Table 2:** Summary of the state-of-the-art comparison with respect to both compressed size and random access time

Method	Compressed size [MiB]	Random access area [Mbp s]
GVC	897	311
GTC	1136	256
GTRAC	2986	N/A${}^\dag$
GTShark	587	N/A${}^*$

${}^*$ Random access is not implemented. ${}^\dag$ Similar to what the authors of GTC report, we were not able to evaluate GTRAC on the present dataset

## Conclusion

We present a novel approach for compressing gene sequence variations: the Genomic Variant Codec (GVC). Better compression is achieved by using techniques such as joint row- and column-wise sorting of blocks of variations and by using the existing image compression standard JBIG for efficient entropy coding. At the same time, GVC allows non-sequential data retrieval by splitting the data into blocks. To find row and column orders that are beneficial in terms of entropy coding, we solved a problem analogous to the traveling salesman problem by using the nearest neighbor algorithm. Other solvers such as the Lin-Kernighan heuristic [12] could be employed to achieve a potentially better compression at the expense of encoding time.

GVC meets the state of the art in terms of compression ratio: our results show that GVC reduces the genotype information size from 758 GiB down to 897 MiB on the publicly available 1000 Genomes Project (phase 3) data, outperforming its competitor GTC, which is only able to reduce the raw data size to 1136 MiB. Note, however, that GTC offers slightly faster random access. With respect to GTShark, GVC does not match its compression performance, but provides, in contrast, random access capability that makes GVC applicable in real-world scenarios. In summary, GVC provides a new solution in the compression-feature space by offering excellent compression combined with random access functionality. GVC could be adapted for other integral sample-level annotations such as the per-allele read depth, alternate allele counts, or quality metrics.

## Data Availability

The 1000 genome project dataset is available at www.internationalgenome.org/data/ or http://ftp.1000genomes.ebi.ac.uk/vol1/ftp/release/20130502/.

## Abbreviations

SNP: Single-nucleotide polymorphism
indels: Insertions and deletions
VCF: Variant call format
GTRAC: GenoType Random Access Compressor
GTC: GenoType Compressor
GVC: Genomic variant codec

## Footnotes

1 Please note that we did not favor JBIG’s successor, JBIG2 (ISO/IEC 14492 [13]), since it does not noticeably increase the performance of lossless compression for this kind of data.

## Code availability

GVC is open source and available at https://github.com/sXperfect/gvc/.
